# Variability in the Perception of Informed Consent for IV-tPA during Telestroke Consultation

**DOI:** 10.3389/fneur.2012.00128

**Published:** 2012-08-27

**Authors:** Lisa Thomas, Anand Viswanathan, Thomas I. Cochrane, John Johnson, Janice O’Brien, Marilyn McMahon, Janine Marie Santimauro, Lee H. Schwamm

**Affiliations:** ^1^Department of Emergency Medicine, Mount Auburn HospitalCambridge, MA, USA; ^2^Department of Neurology, Massachusetts General Hospital and Harvard Medical SchoolBoston, MA, USA; ^3^Department of Neurology, Brigham and Women’s Hospital and Harvard Medical SchoolBoston, MA, USA; ^4^Department of Risk Management, Massachusetts General HospitalBoston, MA, USA

**Keywords:** informed consent, IV-tPA, stroke, telestroke, ischemic stroke patients

## Abstract

**Objective:** To study the perception of informed consent among various raters for thrombolysis in acute ischemic stroke patients receiving intravenous tissue plasminogen activator (IV-tPA). **Methods:** Twenty randomly selected videotaped telestroke consultations of acute stroke patients administered IV-tPA were retrospectively reviewed. Adequacy of informed consent was reviewed by five raters: a neurologist and emergency physician who routinely treat stroke, a medical risk management paralegal, a bioethicist, and a lay person. Raters assessed the quality of the informed consent presentation by the treating physician and the degree of understanding demonstrated by the patient/family authorizing consent. Factors associated with adequacy of consent were analyzed. **Results:** Consent was rated as adequately understood by the patient-family in 78.6% cases. Agreement between all five raters with regard to the patient-family understanding of consent was poor and also between the subgroups of non-physician and physician (all *k* < 0.20). Similarly, the quality of the physician consent process was poor for agreement between all five raters (*k* = 0.07) or between the subgroup of the three non-physician raters (*k* = −0.06) and fair between the two physician raters (*k* = 0.24). The legal reviewer and the bioethicist rated the physician consent process as being of lower quality than did the two physicians and the layperson. **Conclusion:** Despite high variability in the perception of informed consent among raters in this time-sensitive clinical situation, almost 80% of patients were rated by all reviewers as having adequate understanding of risks and benefits of tPA. This suggests the need for a standardized but brief tPA consent process that includes patient/family demonstration of understanding.

## Introduction

Intravenous tissue plasminogen activator (IV-tPA) was approved in 1996 by the FDA for the treatment of acute ischemic stroke within 3 h of symptom onset (FDA Center for Biologics Evaluation and Research, 1996). Although written consent is not required prior to drug administration, the American Heart Association Guidelines recommend that, the potential risks and benefits should be explained to the patient and their family (Adams et al., 2003). These potential benefits of IV-tPA, must be weighed against the potential risks prior to treatment decision (The NINDS t-PA Stroke Study Group, 1995, 1997).

Physicians are ethically obligated to explain these specific issues to the patient (or legally authorized representative if the patient lacks capacity) in plain language that is easily understood. This communication process in which patients receive relevant information related to their diagnosis and treatment options and subsequently make an informed decision to authorize a specific treatment is captured under the domain of “informed consent” (Paterick et al., 2008; Schenker et al., 2011). The key elements of informed consent include disclosure of information including risks, benefits, and alternatives, voluntary choice, and decisional competence (White-Bateman et al., 2007; Schenker et al., 2011). However, in the heat of the moment when every minute of delay reduces the chances of recovery in acute stroke, the informed consent process may become compressed or distorted.

Telemedicine-enabled acute stroke (telestroke) consultation to a network of referring hospitals in New England has been a routine part of acute stroke care for our faculty and fellows for the past decade. In this setting, all telestroke consultations are recorded and so this platform provides an excellent opportunity to study the consent process without the intrusive presence of a camera, tape recorder, or third party analyst distorting the process. These video recordings allow for multiple raters to review the same consent discussions and compare their assessments. We sought to determine the perception of the nature and quality of the consent for IV thrombolysis in real practice, from both the patient and provider perspective by analyzing a series of recorded telestroke consultations.

## Materials and Methods

This was a retrospective review of the informed consent process in acute ischemic stroke patients who received telestroke consultation for administration of IV-tPA. All patients presented to a referring outside hospital within 3 h of symptom onset and were evaluated by video at our tertiary care center. The study was approved by our local Institutional Review Board.

### Telestroke system

The Massachusetts General Hospital TeleStroke program consists of 26 member hospitals in the greater New England area, the details of which have been previously described (Schwamm et al., 2004; Pervez et al., 2010). Briefly, it uses a decision-supported model of live, interactive videoconferencing coupled with remote review of brain images via teleradiology to enable the evaluation of remotely located acute stroke patients for IV-tPA eligibility. Clinical verbal consent is always obtained from the patient or surrogate prior to thrombolysis. The full consultation encounter is recorded in a standard viewable video format, and was made available for review. Patients are consented for participation in the telestroke consultation including for teaching and research purposes related to the telestroke encounter.

### Patient selection and telestroke case review

Video recordings of 20 patients who received IV-tPA after telestroke consultations at six outside hospitals between May 2007 and October 2008 were randomly selected for evaluation by reviewers from 67 patients who received IV-tPA during this period. There were no patients who declined treatment. The physician performing informed consent via TeleStroke consultation varied among the 20 cases. Adequacy of informed consent was assessed by five independent raters: a neurologist (MD1) and emergency physician (MD2) who routinely treat stroke, a medical risk management legal advisor, a bioethicist, and a lay person without medical training or experience in stroke or consent. Reviewers were not involved in the original patient encounter and were blinded to the other reviewers’ ratings and any details about patient outcomes. Furthermore, the study was retrospective in nature and we chose recordings taken prior to any discussion of the proposed study or its design to avoid any contamination of the consent process. In all cases the patient, a legally authorized representative or surrogate, or both, were involved in the consent process. Cases were excluded by the reviewers if the entire consent process was not completely captured on the video (*n* = 3), or if technical limitations in audio or video quality related to patient/family or provider comments during the consent process prevented any reviewers from assigning scores (*n* = 3). Numbered video recordings (in Windows Media Player or QuickTime format) of each consultation were provided to reviewers. These media formats have free viewing software that permit the user to view any section of the recording, and to pause or re-play, as desired. There were no limits placed on how reviewers evaluated the recordings, or how often they reviewed them prior to scoring.

To avoid biasing the reviewers, the following minimal instructions were given rather than a checklist of individual elements of informed consent: “Please watch the following video clips and based on your background and experience rate the quality of consent seen on the video. There are two aspects of the consent to be rated: (1) Did the MD explain the risks and benefits? (2) Did the patient or caregiver appear to understand the risks and benefits? Score each question on the following scale: 0 = no, 1 = yes but needs improvement, 2 = adequate, 3 = exceeds expectations.”

### Outcomes and statistical analysis

As described above, a four-point Likert scale (Likert, 1932) was developed for the purpose of rating two distinct aspects of the consultation process: the quality of informed consent process as presented by the treating physician and the degree of understanding demonstrated by the patient or family member authorizing consent. This ordinal numerical scale (0 = insufficient, 1 = poor, 2 = adequate, and 3 = exceeds expectations) was used to rate all cases. Acceptable understanding of the consent process by the patient or family was defined as occurring when Likert scores were ≥2. We chose an even-point scale in order to construct a forced-choice scale where neutral ratings of the consent process were not possible. When numerical Likert scale scores were grouped across raters, mean values are reported as appropriate. Likert scale scores grouped across cases for each individual rater are reported as median values. Factors potentially associated with adequacy of consent (age, sex, education level, duration of consent, NIHSS, patient, or family providing consent) were analyzed, however sample size was too low to conduct any formal comparative testing. Levels of agreement between raters was determined by un-weighted kappa values, with agreement defined as poor (*k* < 0.20), fair (*k* = 0.20–0.39), moderate (*k* = 0.40–0.59), good (*k* = 0.60–0.79), and excellent (*k* = 0.80–1.0). Statistical analysis was performed using JMP software (version 8, SAS Corporation).

## Results

**Figure 1:**
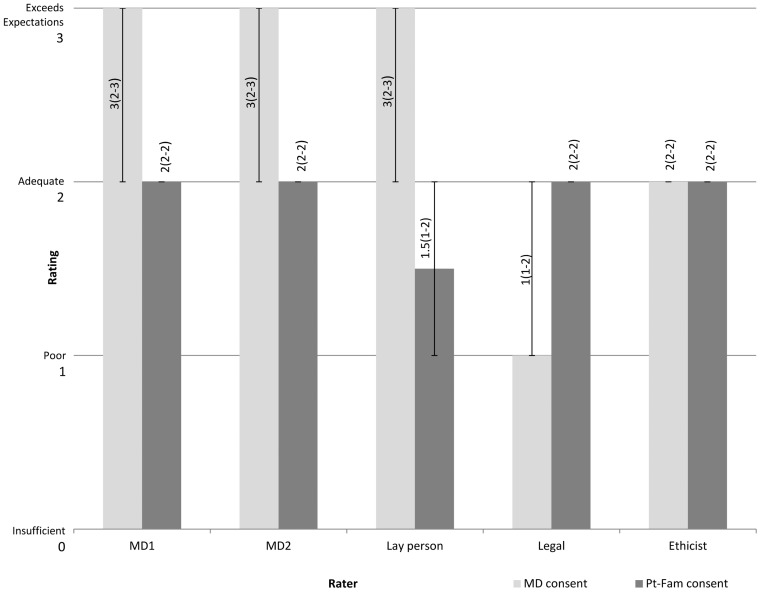
**Distribution of scores among raters**. Median Likert score ratings are shown for five raters. The Likert scale rating consists of an ordinal numerical value ranging from 0 = insufficient, 1 = poor, 2 = adequate to 3 = exceeds expectations. The numbers depicted vertically along the bars show the median Likert score with interquartile range in parenthesis. MD1 is neurologist; MD2 is emergency physician. “MD consent” refers to quality of the consent process provided by the treating physician. “Pt-Fam consent” refers to adequacy of the understanding demonstrated by the patient and/or family authorizing consent.

All patients were English speaking with a mean age of 69 ± 15.8 years and median initial NIHSS score over telestroke of 11.5 points. Family members gave primary consent in 50% of cases. The duration (mean ± SD) of the consent discussion was 2.7 ± 1.3 min. The distribution of scores varied for the two categories by different raters (Figure 1), and there were notable similarities among certain groups of raters.

Acceptable understanding of the consent process occurred in 11/14 (78.6%) cases. In the remaining three cases, the mean of all numerical reviewer Likert scores was <2. There were no differences in baseline characteristics between those subjects in whom patient-family understanding of consent was rated as acceptable vs. unacceptable (Table 1), however, the mean consent duration was longer in the acceptable consent group (2.8 vs. 1.8 min) and the time elapsed from last seen well to thrombolysis tended to be shorter in this group (126.4 vs. 159 min). Acceptable quality of the physician consent process was defined similarly as a mean Likert score ≥2. Compared to those cases in which the treating physician was rated as not providing acceptable consent, there were no differences in patient characteristics (Table 2).

**Table 1:** **Patient characteristics for all patients and patients with acceptable vs. unacceptable patient/family consent**.

	All patients (*n* = 14)	Pt-Fam acceptable consent (*n* = 11)	Pt-Fam unacceptable consent (*n* = 3)
Age (mean years ± SD)	69 ± 15.8	68.8 ± 17.3	69.7 ± 11.1
Female	5 (35.7%)	5 (45.5%)	0 (0%)
High school graduate (*n* = 10)	6 (60%)	4 (50%)	2 (100%)
History of hypertension	12 (85.7%)	9 (81.8%)	3 (100%)
History of diabetes mellitus	3 (21.3%)	2 (18.2%)	1 (33.3%)
History of hyperlipidemia	7 (50%)	4 (36.4%)	3 (100%)
Family present for consent	11 (78.6%)	9 (81.8%)	2 (66.7%)
Person authorizing consent		
Patient	4 (28.6%)	3 (27.3%)	1 (33.3%)
Family	7 (50%)	6 (54.6%)	1 (33.3%)
Both	3 (21.4%)	2 (18.2%)	1 (33.3%)
Duration of consent (minutes ± SD; *n* = 13)	2.7 ± 1.3	2.8 ± 1.3	1.8 ± 1.1
Time last seen well to tPA (minutes ± SD)	133.4 ± 21.9	126.4 ± 17.6	159.0 ± 18.1
NIHSS [median (IQR)]	11.5 (7–17.3)	11 (7–17)	12 (11–18)
NIHSS item 9: best language [median (IQR)]	0.5 (0–2.3)	0 (0–3)	1 (0–2)

**Table 2:** **Patient characteristics for acceptable vs. unacceptable treating MD consent**.

	MD acceptable consent (*n* = 12)	MD unacceptable consent (*n* = 2)
Age (mean years ± SD)	67.9 ± 16.8	75.5 ± 6.36
Female	5 (41.7%)	0 (0%)
High school graduate (*n* = 10)	5 (55.6%)	1 (100%)
History of hypertension	10 (83.3%)	2 (100%)
History of diabetes mellitus	3 (25%)	0 (0%)
History of hyperlipidemia	5 (41.7%)	2 (100%)
Family present for consent	10 (83.8%)	1 (50%)
Person authorizing consent		
Patient	3 (25%)	1 (50%)
Family	6 (50%)	1 (50%)
Both	3 (25%)	0 (0%)
Duration of consent (minutes ± SD; *n* = 13)	2.8 ± 1.3	1.1 ± 0.0
Time last seen well to tPA (minutes ± SD)	130.5 ± 22.0	150.5 ± 14.8
NIHSS [median (IQR)]	11.5 (7–16)	14.5 (11–18)
NIHSS item 9: best language [median (IQR)]	0.5 (0–2.8)	1 (0–2)

The level of agreement between raters with regard to the patient-family understanding of consent, as measured by the kappa statistic, was poor between all five raters, between the subgroup of the three non-physician raters, and between the two physician raters (all *k* < 0.20). Similarly, the quality of the physician consent process was poor for agreement between all five raters (*k* = 0.07) or between the subgroup of the three non-physician raters (*k* = −0.06) and fair between the two physician raters (*k* = 0.24). When the ratings were analyzed as overall groups, regardless of rater subgroups, the median [IQR] ratings of patient-family understanding of consent and physician consent process did not differ (2[2–2] vs. 2[2–3]).

When evaluating the relationship between the quality of patient-family understanding to that of the physician consent process, a pattern was observed. Cases in which patient-family understanding was rated acceptable (*n* = 11) had a corresponding rating of the physician consent process in the acceptable range (mean Likert 2.30) as well, whereas those in which patient-family understanding was rated unacceptable (*n* = 3) had a rating of the physician consent process in the unacceptable range (mean Likert 1.60) as well. This mean Likert scale score difference was 0.70.

There were observed differences between types of raters when the quality of the physician consent process was analyzed by median Likert scores assigned. The legal reviewer (1) and the bioethicist (2) rated the physician consent process as being of lower quality than did the two physicians and the layperson (3). When the quality of patient-family understanding was analyzed in a similar manner, the lay person rated this lower than the other raters (1.5 vs. 2).

## Discussion

The major findings from this study are that there is high variability among raters, both physician and non-physicians, regarding the perceived quality of informed consent for thrombolysis in acute stroke and that the conversation regarding informed consent is generally quite brief (mean duration <3 min). Despite this, almost 80% of patients were rated by all reviewers as having adequate understanding regarding the risks and benefits of thrombolytic therapy.

There is a need to better understand, improve, and standardize the process of informed consent for thrombolysis in acute stroke. Studies of the adequacy of the consent process have largely focused on the extent of the risk explanation by retrospective review of written documentation (Jeyaseelan et al., 2010; Patel et al., 2010; Siddins et al., 2009; Ahmad et al., 2009) and exist mostly in the surgical field. Other studies have used interviews or written questionnaires to focus on the patient’s perceptions of consent, most of which reveal that patients perceive the consent process to be inadequate and often sign consent forms without reading them (Lavelle-Jones et al., 1993; Kay and Siriwardena, 2001). In comparing the consent process for elective vs. emergent procedures, patients undergoing emergent procedures are less likely to have read or understood the consent form and are more likely to report feeling frightened by the consent process (Akkad et al., 2004). Therefore, further improvements in the consent process are needed especially in emergent circumstances (Akkad et al., 2004).

Cognitive capacity related to decision making may be impaired in emergency situations. Studies of patients with acute myocardial infarction involved in experimental trials demonstrate that physical and emotional stress alone may hinder understanding of the consent process (Williams et al., 1997, 2003; Smithline et al., 1999; Agard et al., 2001). A large proportion of acute stroke patients are unable to provide consent for enrollment in experimental acute stroke trials due to acute cognitive impairment (Demarquay et al., 2005; Kane et al., 2006). Additional obstacles of obtaining consent in acute stroke may include cognitive impairment due to the stroke itself. The stroke patient may have impaired communication due to dysarthria or aphasia, may be unaware of the illness due to anosognosia, or may have an altered level of consciousness (White-Bateman et al., 2007; Ciccone et al., 2001). These difficulties compounded with the emergent nature of the illness and need for rapid treatment, make consent challenging, especially in patients with greater cognitive deficits.

Retrospective approaches to studying consent adequacy, most commonly by review of documented risks on the consent form (Ahmad et al., 2009; Siddins et al., 2009; Jeyaseelan et al., 2010; Patel et al., 2010) or by immediate or delayed patient interview or questionnaires (Lavelle-Jones et al., 1993; Kay and Siriwardena, 2001) are limited in gaging the true nature of the consent encounter. Written documentation may not reflect the entire verbal exchange or interaction. Furthermore, patient recall of the consent process may be biased. For acute ischemic stroke, our study is the first to date using real-time unedited videos from telemedicine encounters to review the perceived quality of the informed consent process among various raters. As our service uses telestroke consultation as part of our routine clinical practice and the recording of the consults is an unobtrusive by-product of teleconsultation, these recordings likely reflect true clinical practice in a way that would be difficult or impossible to capture during in-person acute stroke evaluations at our hospital.

Informed consent may be perceived differently by different groups of people. For example, there may be systematic differences in perception between physicians, medico-legal experts, patients, and laypeople as to the adequacy of the risks and benefits as explained by the doctor, or the level of understanding evidenced by the patient or family. Our finding of consistently lower ratings of the physician consent process by the two reviewers with professional training in informed consent doctrine supports this hypothesis. They may hold the physician to a more strict set of ethical or legal standards and may not relax those standards despite the time constraints associated with IV-tPA treatment.

Yet despite rater variability, almost 80% of patient and/or families were rated as having adequate understanding of the consent process. This is in contrast to a previous study regarding informed consent in outpatient practice which reported much lower rates of adequate consent (Braddock et al., 1999). In this study, which analyzed over 1000 non-blinded audio taped patient-physician discussions, only 9% of encounters met the very strict definition of complete informed consent (Braddock et al., 1999). This difference in rates is likely due to differing objectives between studies. The aim of our study was to explore different perspectives on the actual practice of informed consent among reviewers of different medical and non-medical backgrounds rather than asses the rates of adherence to a strict set of defined criteria.

Furthermore, in certain situations, fulfilling all of the elements of informed consent may not be possible or desirable (Hall and Schneider, 2000). Specifically, for acute stroke thrombolysis, a decision to treat must be reached quickly after a briefer version of the consent process. Experience in the stroke clinical trial literature suggests that the use of strict consent criteria, or those that require patient or family signature on a consent form, may exclude a large percentage of potential subjects from trial enrollment (Tu et al., 2004; Demarquay et al., 2005; Kane et al., 2006). Similarly, in clinical practice, the informed consent conversation should convey information in a manner that is succinct but understandable to allow for treatment of the maximal number of potentially eligible patients. Furthermore, some studies have suggested that more complete and formal informed consent processes may not improve patient understanding (Stanley et al., 1998) and may provide more information than the patient desires (Degerliyurt et al., 2010). Due to these considerations, a standardized consent protocol for acute stroke thombolysis may require broad consensus from a multidisciplinary group of experts similar to raters in this study. See Table 3 for a proposed telemedicine consent process based on the authors’ experience at their institution.

**Table 3:** **Sample provider script for obtaining patient’s consent for TPA thrombolysis**.

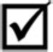 The doctors who have examined you (or your family member) believe that an ischemic stroke is happening right now. This condition occurs when blood flow to the brain is blocked, usually by a blood clot blocking the arteries that deliver blood to the brain. Studies in thousands of patients with strokes like yours have shown that when the clot-dissolving drug alteplase (also called “tpa”) is given intravenously, patients have a much better chance of recovering to normal, and living at home after a stroke. Even though there is a small increased chance of bleeding, the chances of benefit from the treatment are 6–10 times greater than the risks of harm from the treatment. The faster the treatment is started, the better your chances of recovery.
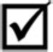 Do you understand what I have explained to you?
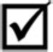 Do you have any questions?
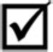 Please use your own words to repeat back to me what I have explained to you. (If there are any misunderstandings, telemedicine physician should correct them and repeat this step.)
□Would you like to proceed with this treatment?
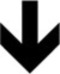
Proceed with thrombolysis

We believe that the ideal tPA consent process should be brief and include the description of an ischemic stroke, the proposed treatment, and the risks associated with that treatment. These elements should be explained clearly and in plain language that is easily understood by the patient and family. There should be a pause to determine whether the patient/family do understand what the provider has explained by asking them to rephrase what they understand. There should also be an opportunity to ask questions. Based on this discussion, a decision to proceed or not proceed with thrombolysis should be made. We do not favor a strict consent criteria as it may be detrimental to the time critical process.

Specific factors adversely affecting quality of informed consent in patients undergoing surgical procedures have been reported, and include older age, lower IQ, and impaired cognition (Lavelle-Jones et al., 1993). We examined a number of potential factors associated with adequacy of consent in stroke thrombolysis, however we did not have the power to detect any differences.

The only known prior study related to the process of informed consent for thrombolytic therapy for acute ischemic stroke focused on the frequency of documentation of informed consent in clinical practice (Rosenbaum et al., 2004). In this retrospective study, a substantial number of patients (15%) had no documented discussion of consent for IV-tPA therapy. Furthermore, surrogates provided consent in 63% of cases where patients still had evidence of capacity for consent (Rosenbaum et al., 2004). This mirrors our findings in that the majority of encounters had some level of family involvement in the consent process. In our study, there were no patients lacking capacity for consent who did not have a surrogate present. Any future standardized consent protocol will likely not apply to the subgroup of these individuals.

Our study has several limitations. We cannot exclude the possibility that our small sample size may have precluded us from detecting associations related to adequacy of consent. We could not assess the level of agreement between raters of the same background because we deliberately chose raters of different training, background, and experience. The level of agreement between the two physicians of different specialties and the two raters with professional training in informed consent suggests that the findings of each rater type might reflect the ratings of that group more broadly, but that will require additional studies. These findings may not be generalizable to other practice settings or consent performed by other providers (non-stroke physicians). There may have been portions of the consent interaction that were not visible on the video footage available to reviewers which may have affected the ratings of quality. We did not randomize the order of video review so it is possible that earlier videos had an effect on the ratings of later viewed videos. Finally, video-based informed consent may differ compared to consent obtained through in-person consultation. Direct comparison of these methods should be performed in future studies.

In summary, high variability exists among reviewers in the perceived quality of informed consent in the time-sensitive clinical setting of thrombolysis for acute stroke. However, despite these challenging circumstances, the majority of patients were rated by all reviewers as having adequate understanding of risks and benefits of IV-tPA treatment. The telestroke consent scenario likely mirrors real-world clinical practice. Although a formal written informed consent is not required for the administration of IV-tPA, this study suggests a potential role for implementing a standardized brief IV-tPA verbal consent process that incorporates a demonstration of patient/family understanding and desire to proceed.

## Conflict of Interest Statement

The authors declare that the research was conducted in the absence of any commercial or financial relationships that could be construed as a potential conflict of interest.
